# Increase in Plasma Oxidized Phosphatidylcholines (OxPCs) in Patients Presenting With ST-Elevation Myocardial Infarction (STEMI)

**DOI:** 10.3389/fmed.2021.716944

**Published:** 2021-12-01

**Authors:** Zahra Solati, Arun Surendran, Andrea Edel, Marynia Roznik, David Allen, Amir Ravandi

**Affiliations:** ^1^Cardiovascular Lipidomics Laboratory, St. Boniface Hospital, Albrechtsen Research Centre, Winnipeg, MB, Canada; ^2^Department of Physiology and Pathophysiology, Rady Faculty of Health Sciences, University of Manitoba, Winnipeg, MB, Canada; ^3^Department of Medicine, Rady Faculty of Health Sciences, University of Manitoba, Winnipeg, MB, Canada; ^4^Section of Cardiology, Department of Medicine, Rady Faculty of Health Sciences, University of Manitoba, Winnipeg, MB, Canada

**Keywords:** reperfusion, lipidomics, oxidized lipid, acute myocardial infarction, percutaneous coronary intervention

## Abstract

**Objective:** ST-segment Elevation Myocardial Infarction (STEMI) occurs as a result of acute occlusion of the coronary artery. Despite successful reperfusion using percutaneous coronary intervention (PCI), a large percentage of myocardial cells die after reperfusion which is recognized as ischemia/reperfusion injury (I/R). Oxidized phosphatidylcholines (OxPCs) are a group of oxidized lipids generated through non-enzymatic oxidation and have pro-inflammatory properties. This study aimed to examine the roles of OxPCs in a clinical setting of myocardial I/R.

**Methods:** Blood samples were collected from STEMI patients at presentation prior to primary PCI (PPCI) (Isch) and at 4 time-points post-PPCI, including 2 h (R-2 h), 24 h (R-24 h), 48 h (R-48 h), and 30 days (R-30 d) post-PPCI. As controls, blood samples were collected from patients with non-obstructive coronary artery disease after diagnostic coronary angiography. Aspiration thrombectomy was also performed in selected STEMI patients. High-performance lipid chromatography-electrospray mass spectrometry (LC-MS/MS) was used for OxPCs analysis.

**Results:** Twenty-two distinct OxPC species were identified and quantified in plasma samples in patients presenting with STEMI. These compounds were categorized as fragmented and non-fragmented species. Total levels of OxPCs did not significantly differ between Isch and control groups. However, total levels of fragmented OxPCs increased significantly in the ischemic period compared with controls (Isch: 4.79 ± 0.94, Control: 1.69 ± 0.19 ng/μl of plasma, *P* < 0.05). Concentrations of non-fragmented OxPCs had significant reductions during ischemia compared to the control group (Isch: 4.84 ± 0.30, Control: 6.6 ± 0.51 ng/μl, *P* < 0.05). Levels of total OxPCs in patients with STEMI were not significantly different during reperfusion periods. However, fragmented OxPCs levels were elevated at 48 h post-reperfusion and decreased at 30 days following MI, when compared to R-2 h and R-24 h time points (Isch: 4.79 ± 0.94, R-2 h: 5.33 ± 1.17, R-24 h: 5.20 ± 1.1, R-48 h: 4.18 ± 1.07, R-30 d: 1.87 ± 0.31 ng/μl, *P* < 0.05). Plasma levels of two fragmented OxPCs, namely, POVPC and PONPC were significantly correlated with peak creatine kinase (CK) levels (*P* < 0.05). As with plasma levels, the dominant OxPC species in coronary aspirated thrombus were fragmented OxPCs, which constituted 77% of total OxPC concentrations.

**Conclusion:** Biologically active fragmented OxPC were elevated in patients presenting with STEMI when compared to controls. PONPC concentrations were subsequently increased after PPCI resulting in reperfusion. Moreover, levels of POVPC and PONPC were also associated with peak CK levels. Since these molecules are potent stimulators for cardiomyocyte cell death, therapeutics attenuating their activities can result in a novel therapeutic pathway for myocardial salvage for patients undergoing reperfusion therapy.

## Highlights

- Fragmented OxPCs with aldehyde group, including POVPC, SOVPC, PONPC and SONPC elevated during ischemia and early reperfusion but decreased over 30 days post-PCI.
- Non-fragmented OxPCs concentrations (namely PAPC-OH, SAPC-OH and isoPGF2alpha-SPC) were significantly elevated during ischemia and 30 days following MI.
- POVPC and PONPC levels during ischemia were associated with peak CK levels, which is a biomarker of tissue injury.
- The OxPC profiles of thrombus and plasma are distinct. The origin of OxPCs in circulation at the onset of a STEMI is not from the coronary thrombus itself.

## Background

Acute myocardial infarction (MI) is one of the leading causes of morbidity and mortality worldwide (1). The restoration of blood flow by the use of thrombolytic agents or percutaneous coronary intervention (PCI) is an effective approach to re-perfuse myocardium, limit infarct size, preserve systolic cardiac function, prevent heart failure and improve mortality (2).

Despite significant reductions in rates of post-MI-mortality and heart failure (HF) over the last 20 years, their incidences are still high (10 and 25%, respectively), which are attributed to ischemia-reperfusion (I/R) injury (3).

I/R injury is defined as myocardial cell death following reperfusion, which is thought to be responsible for 50% of the final infarct size (3). A large component of this ongoing myocardial injury is the result of an extensive production of reactive oxygen species (ROS) and inflammation post-reperfusion. One group of compounds susceptible to oxidation are cellular lipids. Myocardial phosphatidylcholines (PC) are specifically susceptible to oxidation, due to having a high proportion of polyunsaturated fatty acids (PUFA) in their structures. Oxidation of PC molecules at the site(s) of unsaturation can lead to the formation of a variety of OxPC molecules; however, despite similar structures, the acquired biological activities are uncharacteristic of the parent molecule (4). It has been shown that only 4-h treatment of human aortic endothelial cells (HAEC) with 40 μg/ml of oxidized 1-palmitoyl-2-arachidonyl-sn-glycerol-3-phosphocholine (Ox-PAPC) modulates more than 1,000 genes which are implicated in inflammation, angiogenesis, cell division, thrombosis, and vasoconstriction (5).

The role of OxPC in coronary artery disease (CAD) has been explored extensively utilizing the monoclonal antibody E06 (6, 7). E06 is an IgM antibody that binds to OxPCs, but not naive PC (8). OxPCs not only serve as a damage-associated molecular pattern (DAMPs), which are recognized by pattern-recognition receptors (such as TLR2 and/or CD36) but also induce the production of inflammatory cytokines (9). Studies have shown that levels of OxPCs bound to apoB100 increase acutely following ACS (10) and PCI (11). We have recently shown that there are large increases both in cardiomyocytes and myocardial tissue levels of OxPC molecules in both *in vitro* and *in vivo* model of myocardial I/R (12–14). Among the OxPC species, fragmented OxPCs are potent inducers of cell death through a mitochondrial-mediated pathway. We went on to show that inactivating OxPCs resulted in a significant increase in myocardial recovery (14).

Our goal in this study is to identify the plasma OxPC molecules in patients presenting with ST-Elevation Myocardial Infarction (STEMI) undergoing reperfusion in a case-control study. This will allow us to see the temporal changes in these compounds and the impact of reperfusion.

## Materials and Methods

### Materials

Materials are presented in the online Supplementary Materials.

### Study Population (Cases, Controls) and Sample Collection

**Figure 1:**
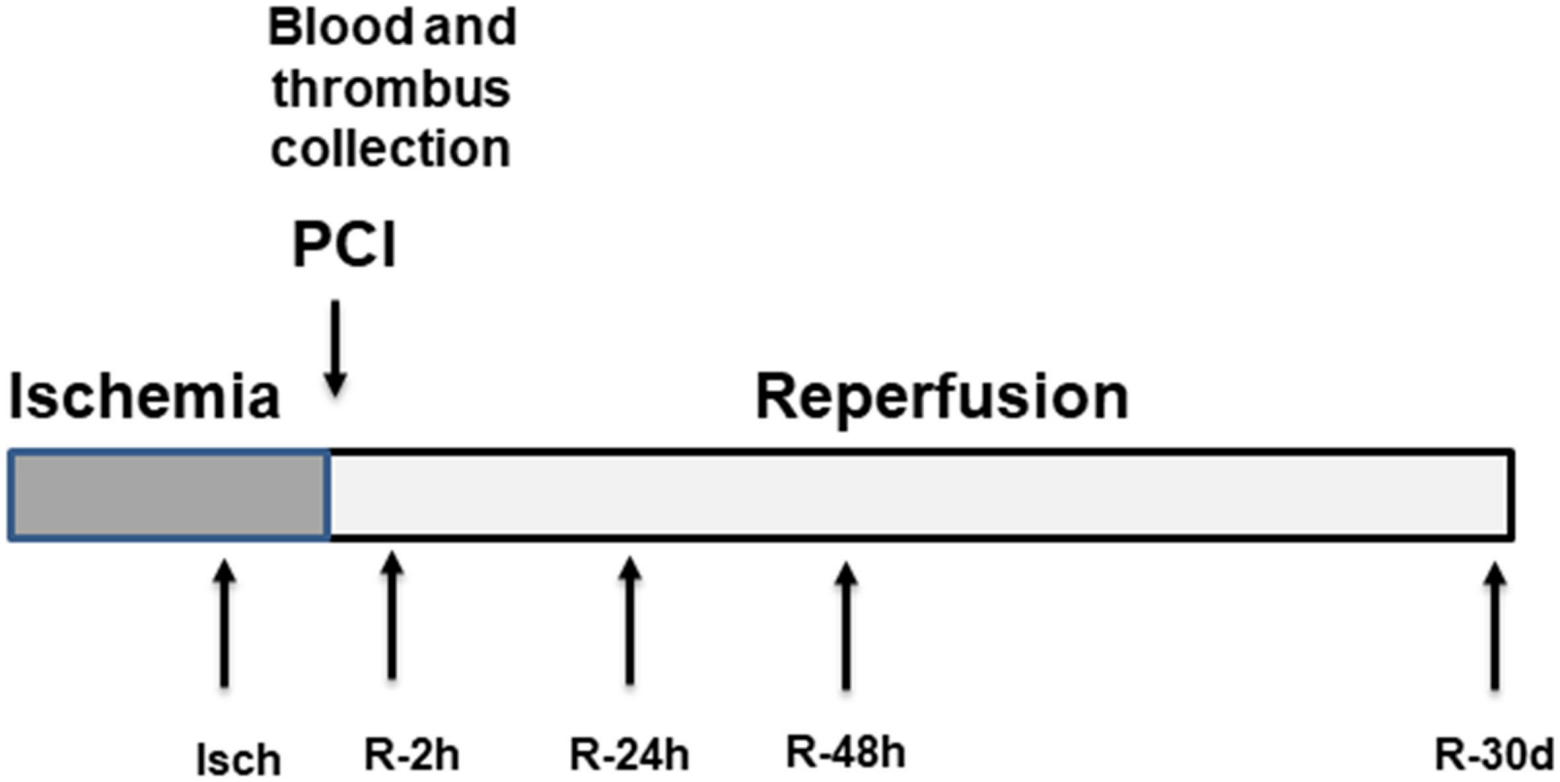
Overall study design. Plasma samples were collected at five different time points including the time of arrival at the cardiac catheterization laboratory for primary PCI (Isch), 2 h post angioplasty (R-2 h), 24 h post angioplasty (R-24 h), 48 h post angioplasty (R-48 h) and 30-day post-PPCI (R-30 d).

All samples were collected at St. Boniface Hospital with the study approval by the University of Manitoba and the St. Boniface Hospital Research ethics boards. Blood samples from 52 STEMI patients were collected at the time of presentation to St. Boniface cardiac catheterization laboratory for PPCI. Written informed consent was collected from all patients. Samples were collected by venipuncture at presentation (Isch), post-procedure after successful PPCI and revascularization (R-2 h), 24 h (R-24 h), 48 h (R-48 h) and 30-day post PPCI (R-30 d). All samples were collected in EDTA venipuncture tubes and immediately centrifuged at 3,000 rpm for 10 min in a refrigerated centrifuge. Plasma was aliquoted in cryovials and frozen at −80 C until analysis. During PPCI, selected groups of patients underwent aspiration thrombectomy (*n* = 15). The recovered martial collected in the filter supplied by the manufacturer was kept in 1 ml of PBS with EDTA and kept at −80 until analysis. The overall study design is shown in Figure 1.

Inclusion criteria included patients >18 years of age, confirmation of STEMI on 12 lead ECG, the presentation with chest pain, no contraindication for collection of 10 ml of blood at the time of the procedure, and documentation of occluded coronary artery with coronary angiography.

For aged-matched controls, blood from patients who were referred for coronary angiography without any evidence of coronary disease, as documented by coronary angiography, was collected post cardiac catheterization. Blood samples used as controls were collected from 59 patients.

### Plasma Oxidized Phospholipids Extraction

Plasma lipid extraction was performed with 2:1 (vol/vol) chloroform: methanol using the method described by Folch et al. (15). The ratio of sample to solvent was 1:10 to achieve optimal extraction (16). Di 9:0 PC (10 ng/μl) was used as an internal standard. A lipid extract was then reconstituted in solvent A (acetonitrile and water 60:40 with 10 mM ammonium formate and 0.1% formic acid) and analyzed by LC-MS/MS (17).

### Thrombus Lipid Extraction

Frozen thrombus samples in PBS-EDTA were thawed on ice and homogenized with the Polytron PT 1,600 E homogenizer until a delicate particulate matter was suspended in PBS-EDTA solution. To prevent heating of the solution, cycles of 20 sec of homogenization and 60 sec on the ice were performed until complete homogenization. The sample was aliquoted and frozen at −80°C until the time of lipid extraction. Homogenized samples were thawed on ice and lipid extraction was performed using the 2:1 (vol/vol) chloroform: methanol. The protocol was adjusted for STEMI thrombus samples to allow 1 ml of sample to be extracted in comparison to 100 μl of a sample as initially described by Folch et al. (15). An internal standard mixture of 9:0 PC (10 ng/μl) was added to each sample before lipid extraction. Similar to plasma samples, a portion of lipid extract was reconstituted in solvent A and analyzed by LC-MS/MS.

The total protein concentration of the homogenate sample was quantified with the Pierce Microplate BCA Protein Assay Kit by Thermo Scientific. Optical Density was read at 570 nm with the Dynex MRX Revelation Microplate Reader. Protein was quantified in micrograms per milliliter of homogenate.

### OxPCs Identification and Quantification

Plasma and thrombus lipid extracts were injected to ZORBAX RRHD Eclipse Plus C18, HPLC column (2.1 × 50 mm, 1.8 μm; Agilent Technology, CA, USA). Gradient elution was performed to separate OxPC species. Solvent A and solvent B were a mixture of Acetonitrile/Water (60:40 vol/vol) and Isopropanol/Acetonitrile (90:10 vol/vol), respectively. Both solvents contained 10 mM ammonium formate and 0.1% formic acid. The time program used was as follows: initial solvent B at 32% until 4.00 min; switched to 45% B; 5.00 min 52% B; 8.00 min 58% B; 11.00 min 66% B; 14.00 min 70% B; 18.00 min 75% B; 21.00 min 97% B; 25.00 min 97% B; 25.10 min 32% B until the elution was stopped at 30.10 min. A flow rate of 0.4 ml/min was used for analysis. The temperature of the column oven and sample tray was 45 and 4°C, respectively.

The HPLC system was coupled to a 4,000 QTRAP^®^ triple quadrupole linear ion trap hybrid mass spectrometer system equipped with a Turbo V electrospray ion source (AB Sciex, Framingham, Massachusetts, USA). Identification of OxPCs was carried out using scheduled Multiple Reaction Monitoring (MRM) using product ion (184.3 m/z, Da), which corresponds to the PC head group. The electrospray ionization voltage and temperature of the ion source were set to 5,500 V and 5,000 C, respectively. High purity nitrogen was used as curtain gas with 26 psi and high purity air was used as nebulizer and heater gas with pressure set at 40 and 30, respectively. The MRM settings were as follows: declustering potential = 125, entrance potential = 10, collision energy = 53, collision cell exit potential = 9 and dwell time = 50 msec. The retention time (RT) window in MRM was set to detect peaks of significance within 60 sec of confirmed retention time and data was collected utilizing Analyst^®^ Software 1.6 (AB Sciex). Multi-quant^®^ Software 2.1 (AB Sciex) was used to compare peak areas of internal standards and unknown analytes to quantitate the results.

OxPC standards including POVPC, PAzPC, PONPC, PGPC, KOdiA-PC, and KDdiA-PC were injected to HPLC-MS/MS first to find retention find (RT) for these standards (Supplementary Figure I). To find RTs for other OxPC species with no available commercial standards, phospholipids standards including PAPC, SAPC, PLPC, SLPC, PDHPC and SDHPC were undergone air oxidation to produce a pool of fragmented and non-fragmented OxPC species derived from these phospholipids as previously described (16, 17). Thin evaporated layers of standard phospholipids in separate test tubes were exposed to air for 2, 6, 12, and 24 h. Non-fragmented OxPCs are produced after a short period of air oxidation (2–6 h) vs. fragmented species obtained following a longer period of oxidation (12–24 h). A mixture of air oxidized lipids (constituted of fragmented, non-fragmented and non-oxidized lipids) was then reconstituted in Sol A and injected into HPLC-MS/MS (16, 17). Even though close to 84 OxPC can be identified in plasma, we selected compounds with levels 5 x above baseline and reproducibility in all plasma samples were used. By using this method, we were able to identify 22 OxPC species in human plasma (Supplementary Table I).

### Statistical Analysis

Data were analyzed using Origin (pro), (version17). OriginLab Corporation, Northampton, MA, USA. One-way analysis of variance (ANOVA) with a Fisher *post-hoc* test for multiple comparisons was used to determine statistical significance between study groups when values where normally distributed. For data that was not normally distributed non-parametric testing of more than two groups, the Kruskal-Wallis test was done. All data are presented as mean ± SEM. *P* < 0.05 was considered statistically significant.

## Results

Characteristics of controls and STEMI patients along with laboratory data, and cardiac markers, including creatine kinase (CK) and high sensitivity troponin (TnT) are presented in Table 1.

**Table 1:** Characteristics of study participants.

**Characteristics**	**STEMI patients** **(*n* = 52)**	**Controls** **(*n* = 59)**	***P*-Value**
Male %	66.6	56.3	0.08
Age, yr (mean ± SEM)	65.2 ± 2.08	60.2 ± 1.49	0.06
Body mass index (BMI) (mean ± SEM)	25.8 ± 1.14	30.2 ± 1.05	0.01^*^
LVEF %	62%	–	
Time (min), onset of chest pain to reperfusion (Median)	150 (52–738)	NA	
LAD Infarct (%)	41.6	NA	
RCA Infarct (%)	50	NA	
Circumflex Infarct (%)	8	NA	
Peak CK (Median) (units/L)	1,105 (141–10,655)	NA	
Peak Tnt (Median) (ng/L)	1,093 (1–10,000)	NA	
**Co-morbidity (%)**			
Type 2 diabetes mellitus	20.8	14.5	0.4
Smoker	12.7	18.7	0.3
Hypertension	41.6	48.1	0.5
Dyslipidemia	41.6	25.9	0.08
**Lipids**			
TG (mmol/l)	1.7 ± 1.4	–	
HDL (mmol/l)	1.2 ± 0.4	–	
LDL (mmol/l)	2.8 ± 0.9	–	
TC (mmol/l)	4.2 ± 1.2	–	
**Medications at baseline (%)**			
ACEI/ARB	20.8	21.8	0.9
Betablocker	6.2	16.3	0.1
Statin	16.6	14.5	0.7

* *significantly different compared with controls (P < 0.05)*.

In this study, 66.6% of the STEMI population and 56.3% of the controls were male (*P* = 0.08). The mean age was 65.2 ± 2.08 in the STEMI population and 60.2 ± 1.49 in controls (*P* = 0.06). The average body mass index (BMI) was significantly different between STEMI and controls populations (25.8 ± 1.14 and 30.2 ± 1.05, respectively) (*P* = 0.005). Based on the laboratory data, the STEMI patients had normal triglycerides (TG), cholesterol (TC), low and high-density lipoprotein (LDL and HDL). There were no significant differences regarding acetylsalicylic acid (ASA), angiotensin-converting enzyme inhibitors/ angiotensin-receptor blockers (ACEI/ARB), beta-blockers and statins use between STEMI and control groups (Table 1).

The median ischemic period (from the onset of chest pain to reperfusion) was 150 min. Fifty per cent of participants had a right coronary artery (RCA) infarct, whereas 41.6% and 8% had left anterior descending coronary artery (LAD) and circumflex coronary artery occlusions, respectively. The prevalence of type 2 diabetes (20.8%), hypertension (41.6%) and dyslipidemia (41.6%) at presentation to the hospital were not significantly different in comparison with controls (Table 1). The prevalence of these chronic diseases was similar to the previous study of STEMI populations (13).

### Identified OxPCs in STEMI Patients and Controls

**Figure 2:**
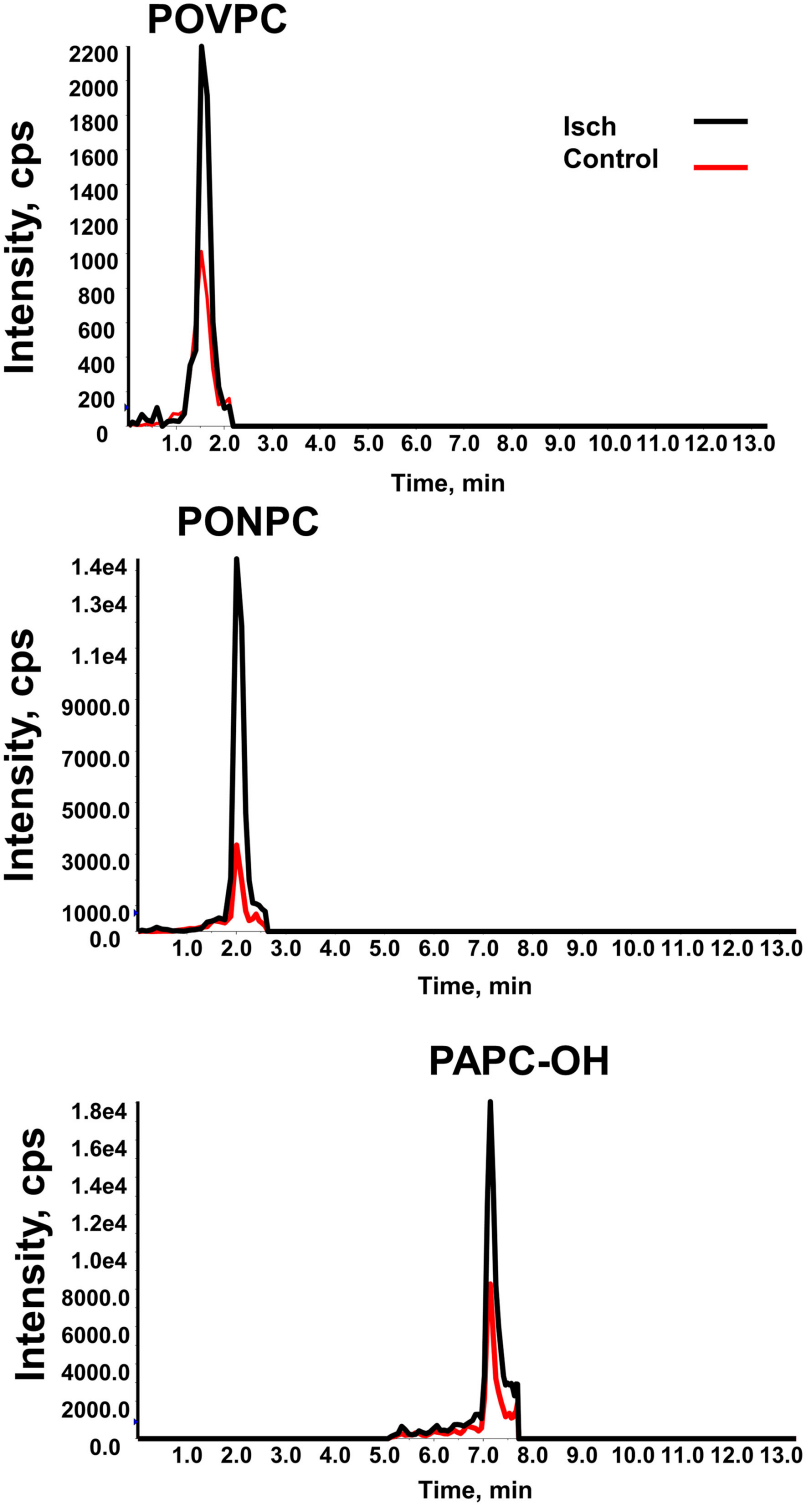
MRM chromatogram of OxPC species. MRM chromatograms of POVPC, PONPC and PAPC-OH in STEMI patients (black line) and controls (red line) measured by reverse-phase LC-MS/MS.

Twenty-two distinct OxPCs were quantified in the plasma of STEMI patients using LC/MS/MS, which include 8 aldehyde-containing OxPC (aldo-OxPC), 6 carboxylic acid-containing OxPC (acid-OxPC) and 8 non-fragmented OxPCs with hydroxyl and hydroperoxyl groups as well as isoprostanes (Table 2). The chromatograms of POVPC, PONPC and PAPC-OH, which are among the highest OxPC species in human plasma, are presented in both ischemia and control groups as examples (Figure 2). Multiple reaction monitoring (MRM) transitions of other identified OxPC compounds are presented in Supplementary Table I.

**Table 2:** Average concentrations of identified OxPCs in study groups.

**OxPC species** **[MH]+/PC[[M-H]+)**	**OxPC categories**	**Control** **(*n* = 59)** **(ng/μl)**	**Isch** **(*n* = 52) (ng/μl)**	**R-2 h** **(*n* = 52)** **(ng/μl)**	**R-24 h** **(*n* = 52)** **(ng/μl)**	**R-48 h** **(*n* = 51)** **(ng/μl)**	**R-30 d** **(*n* = 30)** **(ng/μl)**
4-oxo-butyryl-PC (580.6/184.3)	Aldo-OxPC	0.07 ± 0.00	0.11 ± 0.01	0.11 ± 0.01	0.13 ± 0.02	0.11 ± 0.02	0.08 ± 0.02
POVPC (524.6/184.3)		0.29 ± 0.02	0.44 ± 0.06	0.46 ± 0.08	0.53 ± 0.09	0.42 ± 0.09	0.29 ± 0.04
SOVPC (622.6/184.3)^*^		0.05 ± 0.00	0.24 ± 0.05	0.25 ± 0.05	0.29 ± 0.07	0.24 ± 0.07	0.12 ± 0.02
8-oxo-octanoyl-PPC (636.6/ 184.3)		0.02 ± 0.00	0.18 ± 0.04	0.2 ± 0.05	0.22 ± 0.05	0.19 ± 0.06	0.07 ± 0.02
PONPC (650.6/184.3)^*^		0.48 ± 0.08	1.88 ± 0.39	2.33 ± 0.5	2.36 ± 0.5	1.98 ± 0.5	0.52 ± 0.13
KOOA-SPC (676.6/184.3)		0.07 ± 0.01	0.06 ± 0.01	0.05 ± 0.02	0.04 ± 0.01	0.06 ± 0.02	0.07 ± 0.02
SONPC (678.6/184.3)^*^		0.16 ± 0.03	0.57 ± 0.13	0.69 ± 0.17	0.68 ± 0.17	0.60 ± 19	0.13 ± 0.03
HODA-PPC (706.6/184.3)		0.00 ± 0.00	0.01 ± 0.00	0.01 ± 0.00	0.02 ± 0.00	0.01 ± 0.00	0.00 ± 0.00
Succinoyl-PPC (526.6/184.3)	Acid-OxPC	0.17 ± 0.00	0.40 ± 0.07	0.33 ± 0.05	0.18 ± 0.02	0.15 ± 0.01	0.27 ± 0.03
PGPC (610.6/184.3)		0.21 ± 0.02	0.15 ± 0.02	0.14 ± 0.02	0.13 ± 0.02	0.10 ± 0.01	0.12 ± 0.01
SGPC (638.6/184.3)		0.00 ± 0.00	0.02 ± 0.00	0.02 ± 0.00	0.02 ± 0.00	0.03 ± 0.00	0.03 ± 0.01
KOdiA-PC (664.6/184.3)		0.07 ± 0.01	0.05 ± 0.01	0.05 ± 0.01	0.04 ± 0.01	0.04 ± 0.00	0.05 ± 0.01
PAzPC (666.6/184.3)		0.06 ± 0.01	0.66 ± 0.4	0.60 ± 0.3	0.49 ± 0.2	0.21 ± 0.08	0.09 ± 0.01
HDdiA-PPC (722.6/184.3)		0.00 ± 0.00	0.01 ± 0.00	0.02 ± 0.00	0.02 ± 0.00	0.02 ± 0.00	0.00 ± 0.00
PLPC-keto (772.6/184.3)	Non-fragmented OxPC	0.39 ± 0.11	0.25 ± 0.04	0.35 ± 0.08	0.24 ± 0.04	0.28 ± 0.08	0.18 ± 0.02
PLPC-OH (774.6/184.3)		0.47 ± 0.09	0.60 ± 0.09	0.64 ± 0.12	0.50 ± 0.13	0.44 ± 0.12	0.36 ± 0.06
PAPC-OH (798.6/184.3)^*^		2.00 ± 0.2	1.13 ± 0.1	1.16 ± 0.15	1.54 ± 0.19	1.88 ± 0.2	2.66 ± 0.3
PLPC-OOH,OH (806.6/184.3)		0.39 ± 0.04	0.66 ± 0.11	0.61 ± 0.08	0.52 ± 0.07	0.36 ± 0.05	0.36 ± 0.06
SLPC-epoxy,keto (816.6/184.3)		0.10 ± 0.03	0.15 ± 0.03	0.17 ± 0.04	0.16 ± 0.03	0.14 ± 0.03	0.09 ± 0.02
SAPC-OH (826.6/184.3)^*^		0.50 ± 0.07	0.34 ± 0.03	0.40 ± 0.04	0.61 ± 0.09	0.63 ± 0.1	0.69 ± 0.1
IsoPGF2alpha-PPC (832.6/184.3)^*^		2.36 ± 0.2	1.50 ± 0.1	1.61 ± 0.2	1.27 ± 0.1	1.10 ± 0.1	1.54 ± 0.3
IsoPGF2alpha-SPC (860.6/184.3)^*^		0.40 ± 0.02	0.22 ± 0.02	0.22 ± 0.02	0.20 ± 0.02	0.19 ± 0.02	0.31 ± 0.03

*Data presented as Mean ± SEM*,

* *significantly different among study groups (P < 0.05)*.

### Plasma OxPCs Levels in STEMI Patients During Ischemia Compared With Controls

**Figure 3:**
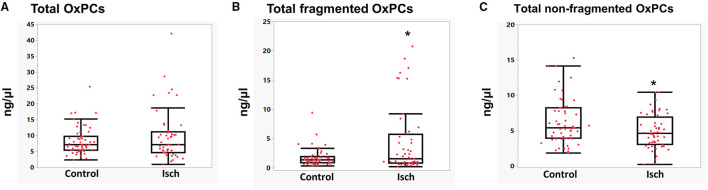
Levels of total OxPCs **(A)**, fragmented **(B)**, and non-fragmented OxPC **(C)** in STEMI patient during ischemia and controls. *Significant differences compared with controls, *P* < 0.05.

Total OxPCs levels did not significantly differ between Isch and control groups (Isch: 9.63 ± 1.08, control: 8.31 ± 0.54) (Figure 3A). However, total levels of fragmented OxPCs were significantly elevated during ischemia in STEMI patients compared with control (Isch: 4.79 ± 0.94, control: 1.69 ± 0.19, *P* < 0.05) (Figure 3B). Total levels of non-fragmented OxPCs were significantly lower in STEMI patients during the ischemia when compared to controls (Isch: 4.84 ± 0.30, control: 6.6 ± 0.51 ng/μl, *P* < 0.05) (Figure 3C).

**Figure 4:**
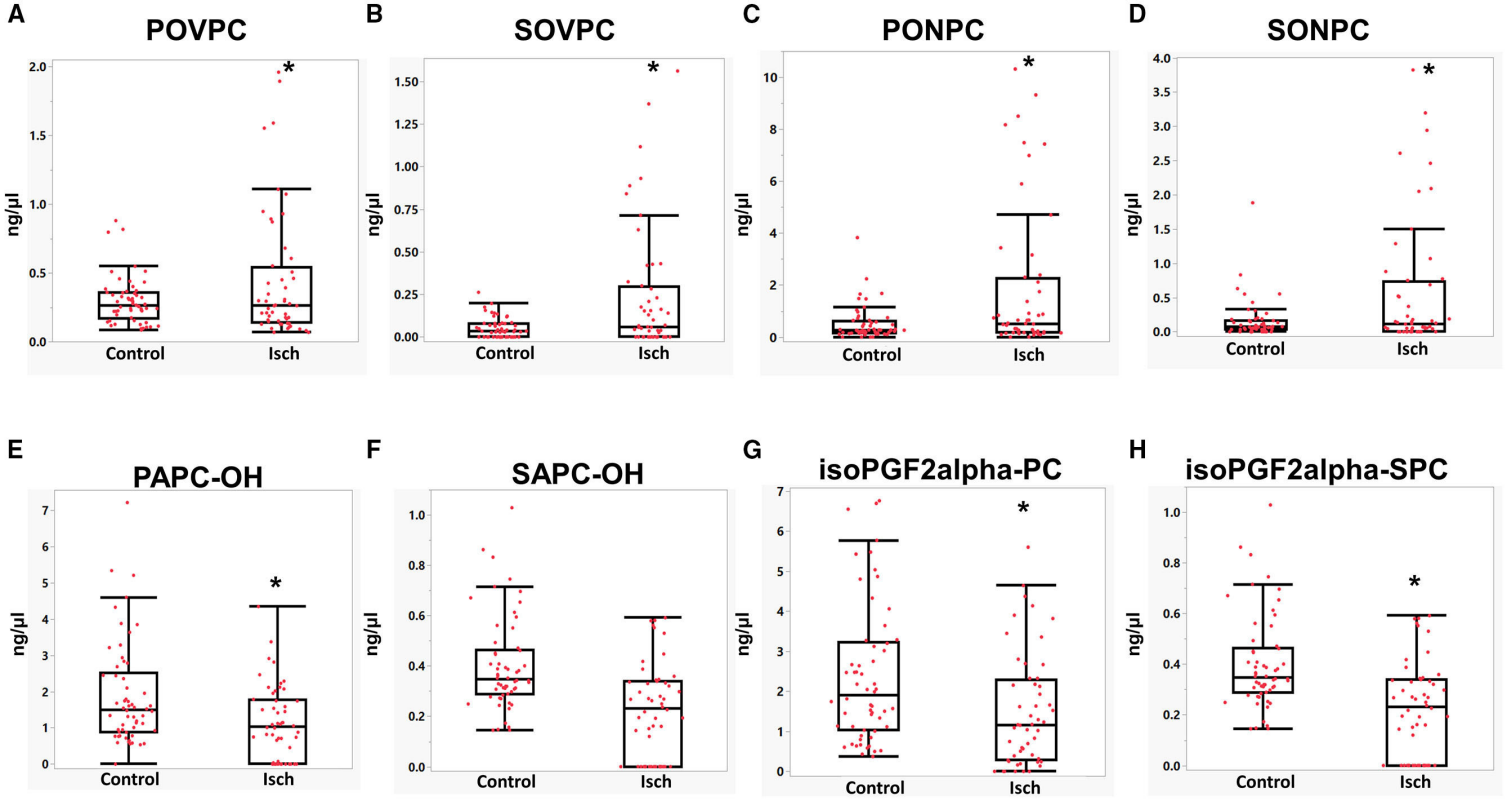
**(A–H)** Mean comparisons of OxPC species in STEMI patient during ischemia and controls. *Significant differences compared with controls, *P* < 0.05.

Looking at individual OxPC species, 4 out of 8 identified aldo-OxPC species, namely POVPC, SOVPC, PONPC, and SONPC, were significantly elevated in Isch when compared to control groups. POVPC concentrations were significantly higher in the Isch group compared with the control (Isch: 0.44 ± 0.06, control: 0.29 ± 0.02 ng/μl, *P* < 0.05) (Figure 4A). As it is shown in Figure 4B, the levels of SOVPC are significantly elevated during ischemia compared with controls (from 0.05 ± 0.01 in controls to 0.2 ± 0.05 ng/μl in Isch *P* = 0.01), which was statistically significant. PONPC levels, which were the most abundant OxPCs in STEMI patients, were significantly higher among STEMI patients when compared to control. The average levels of PONPC were 0.48 ± 0.08 ng/μl in controls compared to 1.87 ± 0.39 ng/μl in the ischemic period in STEMI patients (*P* = 0.02) (Figure 4C). Also, the average levels of SONPC were significantly higher during ischemia compared with controls (Isch: 0.57 ± 0.13 vs. controls: 0.15 ± 0.03 ng/μl, *P* = 0.03) (Figure 4D).

As for non-fragmented OxPCs, 3 out of 8 non-fragmented OxPCs differed significantly in STEMI patients before-PCI compared with controls. The average levels of PAPC-OH reduced significantly in ischemia compared with controls (Isch: 1.13 ± 0.14, control: 2.00 ± 0.2 ng/μl, *P* < 0.05) (Figure 4E). Plasma levels of SAPC-OH were also lower in ischemia compared with controls, although they did not reach statistical significance (Isch: 0.33 ± 0.03 and control: 0.49 ± 0.07 ng/μl of plasma, *P* = 0.08) (Figure 4F). Levels of IsoPGF2alpha-PPC and IsoPGF2alpha-SPC were also significantly elevated in controls compared with Isch group (IsoPGF2alpha-PPC: Isch: 1.49 ± 0.1, control: 2.35 ± 0.2, ng/μl, *P* < 0.05) (Figure 4G) (IsoPGF2alpha-SPC: Isch:0.2 ± 0.02, control: 0.4 ± 0.02, *P* < 0.05) (Figure 4H).

### Changes in Plasma OxPCs Levels in STEMI Patients During I/R

**Figure 5:**
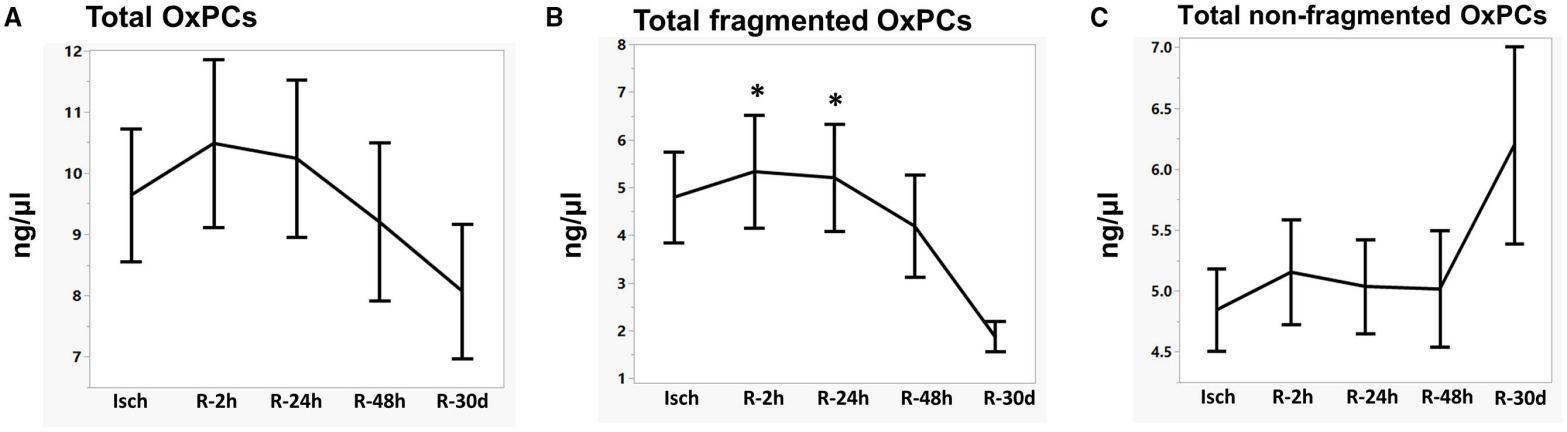
Levels of total OxPCs **(A)**, fragmented **(B)**, and non-fragmented OxPCs **(C)** in STEMI patient during I/R. *Significantly different compared with R-30 d, *P* < 0.05.

We also wanted to assess the changes in OxPC levels during the reperfusion phase compared to the ischemia. Total levels of OxPCs, which constitute both fragmented and non-fragmented OxPCs were not significantly different in STEMI patients during I/R (Isch: 9.63 ± 1.08, R-2 h: 10.48 ± 1.37, R-24 h: 10.24 ± 1.27, R-48 h: 9.20 ± 1.29, and R-30 d: 8.07 ± 1.09 ng/μl) (Table 3; Figure 5A). However, levels of fragmented OxPCs remained elevatored during 48 h post-PCI and then decreased during 30 days of reperfusion, which was statistically significant compared to the levels of Isch and R-2 h groups (Isch: 4.79 ± 0.94, R-2 h: 5.33 ± 1.17, R-24 h: 5.20 ± 1.11, R-48 h: 4.18 ± 1.07, R-30 d: 1.87 ± 0.31 ng/μl, *P* < 0.05) (Table 3) (Figure 5B). In contrast, total levels of non-fragmented OxPCs increased during 30 days after reperfusion, although changes were not statistically significant (Isch: 4.84 ± 0.30, R-2 h: 5.15 ± 0.40, R-24 h: 5.03 ± 0.37, R-48 h: 5.01 ± 0.47, R-30 d: 6.19 ± 0.80) (Table 3; Figure 5C).

**Table 3:** Average levels of total OxPCs, fragmented and non-fragmented OxPCs in study groups.

**OxPC categories (ng/μl)**	**Control** **(*n* = 59)**	**Isch** **(*n* = 52)**	**R-2 h** **(*n* = 52)**	**R-24 h** **(*n* = 52)**	**R-48 h** **(*n* = 51)**	**R-30 d** **(*n* = 30)**
Total OxPC	8.31 ± 0.54	9.63 ± 1.08	10.48 ± 1.37	10.24 ± 1.27	9.20 ± 1.29	8.07 ± 1.09
Total fragmented OxPC	1.69 ± 0.19	4.79 ± 0.94^*^	5.33 ± 1.17^†^	5.20 ± 1.11^†^	4.18 ± 1.07	1.87 ± 0.31
Total non-fragmented OxPC	6.6 ± 0.51	4.84 ± 0.30^*^	5.15 ± 0.40	5.03 ± 0.37	5.01 ± 0.47	6.19 ± 0.80

* *significantly different compared with controls (P < 0.05)*,

† *significantly different compared with R-30 d, (P < 0.05)*.

**Figure 6:**
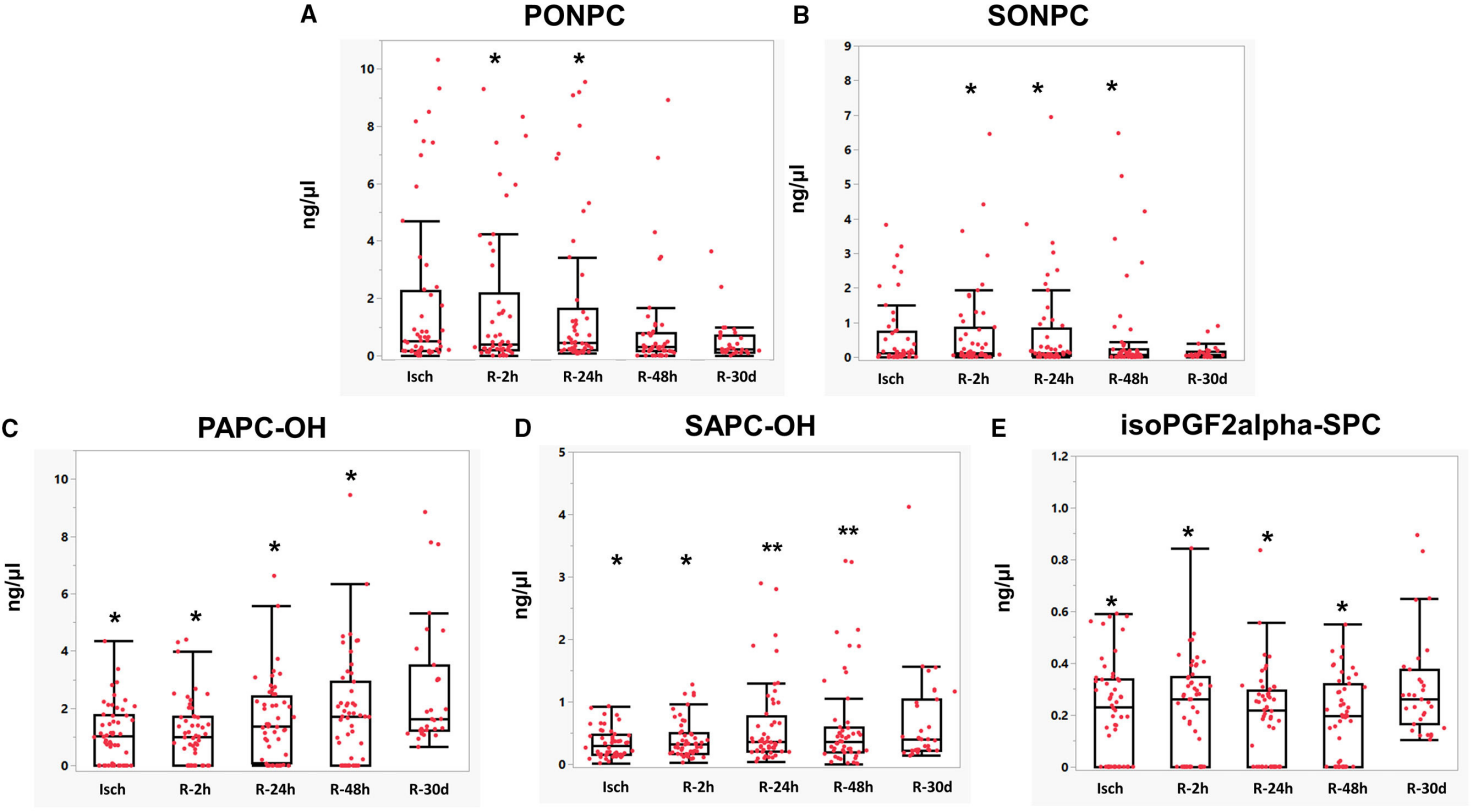
**(A–E)** Mean comparisons of OxPC species in STEMI patient during I/R. *Significantly different compared with R-30 d, *P* < 0.05. **Significantly different compared with the Isch group.

Among fragmented OxPC species, levels of PONPC decreased significantly in 30 day after reperfusion, which was significant compared with R-2 h and R-24 h groups (Isch: 1.87 ± 0.39, R-2 h: 2.3 ± 0.55, R-24 h: 2.36 ± 0.55, R-48 h: 1.98 ± 0.57, and R-30 d: 0.5 ± 0.1 ng/μl, *P* < 0.05) (Figure 6A). SONPC concentrations also decreased over 30 days and changes were statistically significant compared with R-2 h, R-24 h and R-48 h groups (Isch: 0.57 ± 0.13, R-2 h: 0.69 ± 0.13, R-24 h: 0.68 ± 0.17, R-48 h:0.6 ± 0.19, and R-30 d: 0.12 ± 0.03 ng/μl of plasma) (R-2 h and R-30 d: *P* = 0.01, R-24 h and R-30 d: *P* = 0.01, and R-48 h and R-30 d: *P* = 0.04) (Figure 6B).

Non-fragmented OxPC species generally increased over 30 days post-PCI. PAPC-OH levels started to elevate after 24 h of reperfusion. At 30-day post-reperfusion, PAPC-OH levels were at the highest levels, which were statistically significant compared with all other groups (Isch: 1.12 ± 0.14, R-2 h: 1.16 ± 0.15, R-24 h: 1.54 ± 0.19, R-48 h: 1.88 ± 0.2 and R-30 d: 2.66 ± 0.39 ng/μl, *P* < 0.05) (Figure 6C). Levels of SAPC-OH also rose post-reperfusion. It's levels at 24 h and 48 h post-reperfusion were significantly higher compared with ischemic levels (Isch: 0.33 ± 0.03 and R-24 h: 0.60 ± 0.09 ng/μl, *P* = 0.01) (Isch: 0.33 ± 0.03 and R-48 h: 0.62 ± 0.1 ng/μl, *P* = 0.01). SAPC-OH levels continued to increase and its concentrations were significantly elevated at 30-day post-MI in comparison with Isch and R-2 h groups (Isch: 0.33 ± 0.03, R-2 h: 0.40 ± 0.04, R-30 d: 0.69 ± 0.14, *P* < 0.05) (Figure 6D). Levels of isoPGF2alpha-SPC were significantly elevated at 30 days post -MI compared with other STEMI groups (Isch:0.2 ± 0.02, R-2 h: 0.2 ± 0.02, R-24 h: 0.19 ± 0.02, R-48 h: 0.19 ± 0.02, and R-30 d: 0.3 ± 0.03 ng/μl, *P* < 0.05) (Figure 6E). Changes in the levels of all identified OxPCs are presented in Table 2.

### OxPCs Levels and Markers of Myocardial Injury

**Figure 7:**
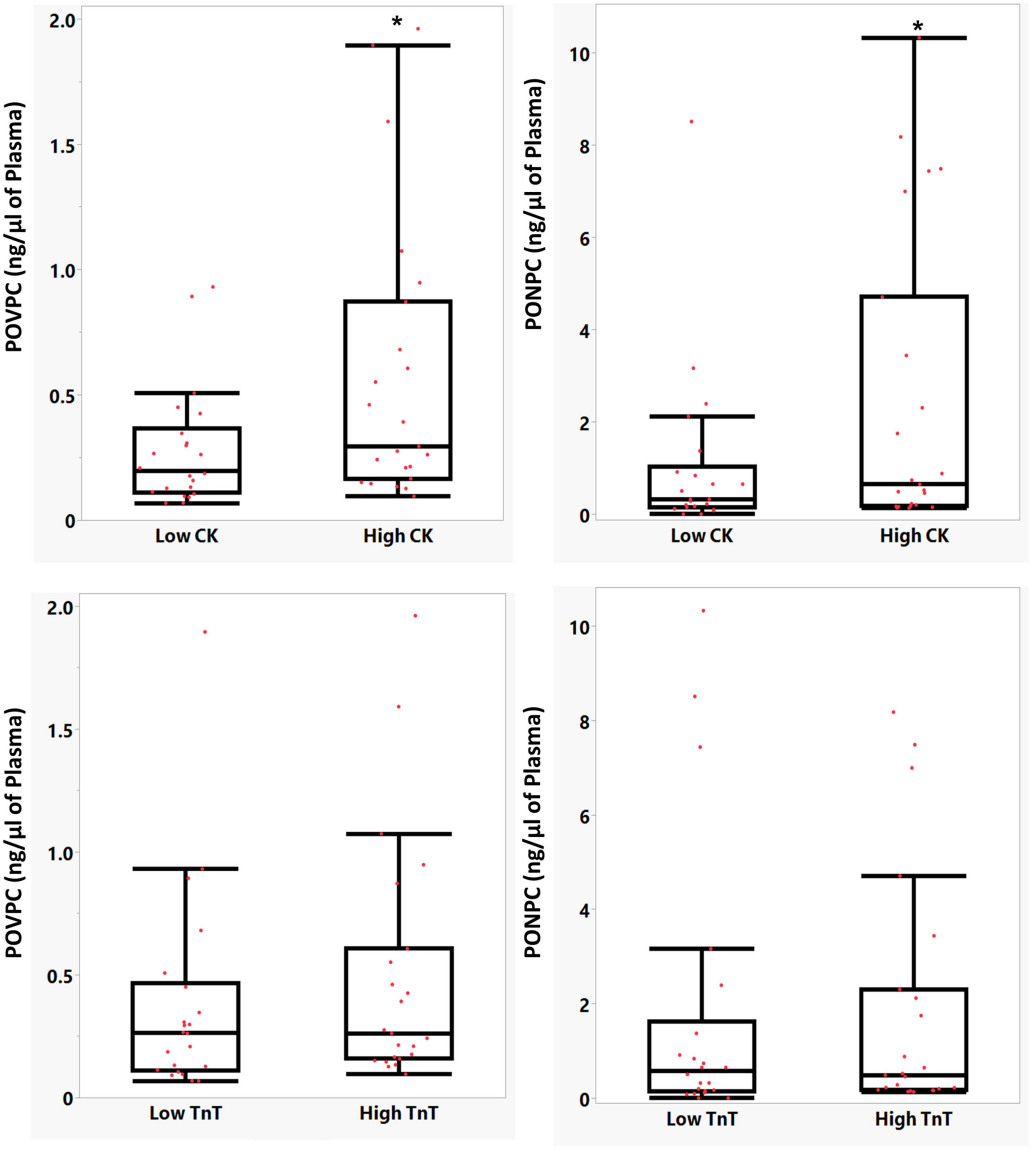
Mean comparison of POVPC and PONPC levels in two groups based on CK **(A)** and TnT levels **(B)**. *Statistically significant difference, *P* < 0.05.

We categorized STEMI patients based on plasma peak CK and TnT levels, which are the gold standards of myocardial injury (18). Patients with levels ≤ than median peak levels of CK were grouped as “low CK” group and patients with levels of > median peak CK levels considered as “high CK” group. As shown in Figure 7A, the levels of POVPC and PONPC in STEMI patients during ischemia were significantly higher in the “high CK” group compared with the “low CK” group (*P* < 0.05). Categorizing POVPC and PONPC based on peak TnT levels also resulted in similar findings meaning that patients with higher TnT levels had higher POVPC and PONPC before PCI. However, differences between TnT groups were not statistically significant (Figure 7B).

### Analysis of OxPCs in Thrombus

In order to see similarities in OxPC species in thrombus material to the plasma, OxPC levels were determined in recovered thrombectomy samples from 15 STEMI patients. Fragmented (aldo/acid) OxPCs and non-fragmented OxPCs containing terminal furans, isoprostanes and long-chain groups were identified in thrombectomy samples. All quantified OxPC species in thrombus are presented in Table 4.

**Table 4:** Quantified OxPCs in thrombus of STEMI patients.

**Component name**	**Average ± SEM (pg/μg protein)**	**Percentage** **of total** **OxPC**
PONPC	1.00 ± 0.18	19.81
POVPC	1.26 ± 0.33	18.48
Isofuran-PC	0.67 ± 0.15	9.90
SOVPC	0.60 ± 0.19	8.78
PAzPC	0.45 ± 0.07	6.65
4-oxo-butyryl-PC	0.38 ± 0.13	5.54
PLPC-epoxy, ketoPLPC-OH, keto	0.36 ± 0.00	5.31
PGPC	0 ± 0.17	3.64
PLPC-OOH,OH	0.25 ± 0.04	3.29
SGPC	0.22 ± 0.07	3.28
SONPC	0.22 ± 0.07	1.66
PLPC-OOH	0.11 ± 0.02	1.57
Succinoyl-PC	0.10 ± 0.03	1.53
Acetal-POVPC	0.10 ± 0.01	1.24
PLPC-OH	0.08 ± 0.025	1.14
SLPC-OOH,OH,keto	0.07 ± 0.03	1.03
SAzPC	0.07 ± 0.02	1.01
KDiA-SPC	0.06 ± 0.01	0.87
PLPC-keto	0.05 ± 0.04	0.71
Furylbutanoyl-PC	0.04 ± 0.008	0.71
PAPC-OH	0.04 ± 0.03	0.56
Furyloctanoyl-PC	0.03 ± 0.01	0.46
KDdiA-PC	0.03 ± 0.01	0.45
HDiA-PC	0.03 ± 0.02	0.45
KOHA-PC	0.03 ± 0.02	0.31
KODA-PPC	0.02 ± 0.02	0.30
SLPC-keto	0.02 ± 0.007	0.27
HODA-PPC	0.01 ± 0.007	0.23
HDdiA-PPC	0.01 ± 0.01	0.21
10-OH-5,8,11-tridecatrienoyl-PC	0.01 ± 0.01	0.18
HODA-SPC	0.01 ± 0.00	0.12
isoPG(A2,J2)-SPC	0.00 ± 0.009	0.10
KOdiA-PC	0.008 ± 0.006	0.03
Acetal-PONPC	0.006 ± 0.006	0.01
Acetal-SONPC	0.002 ± 0.002	0.01

**Figure 8:**
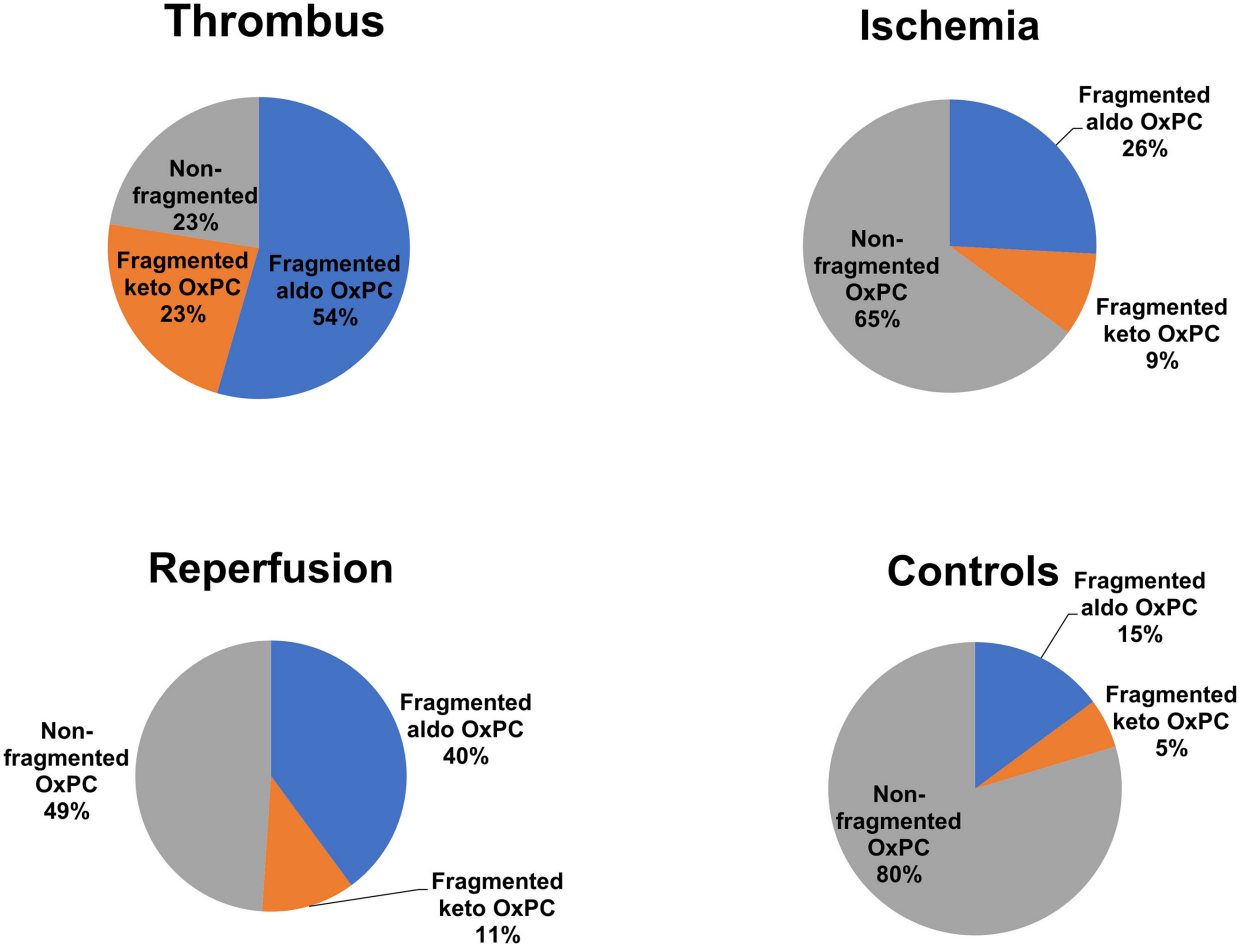
Percentage of fragmented and non-fragmented OxPCs to total OxPCs in thrombus and plasma during I/R.

**Figure 9:**
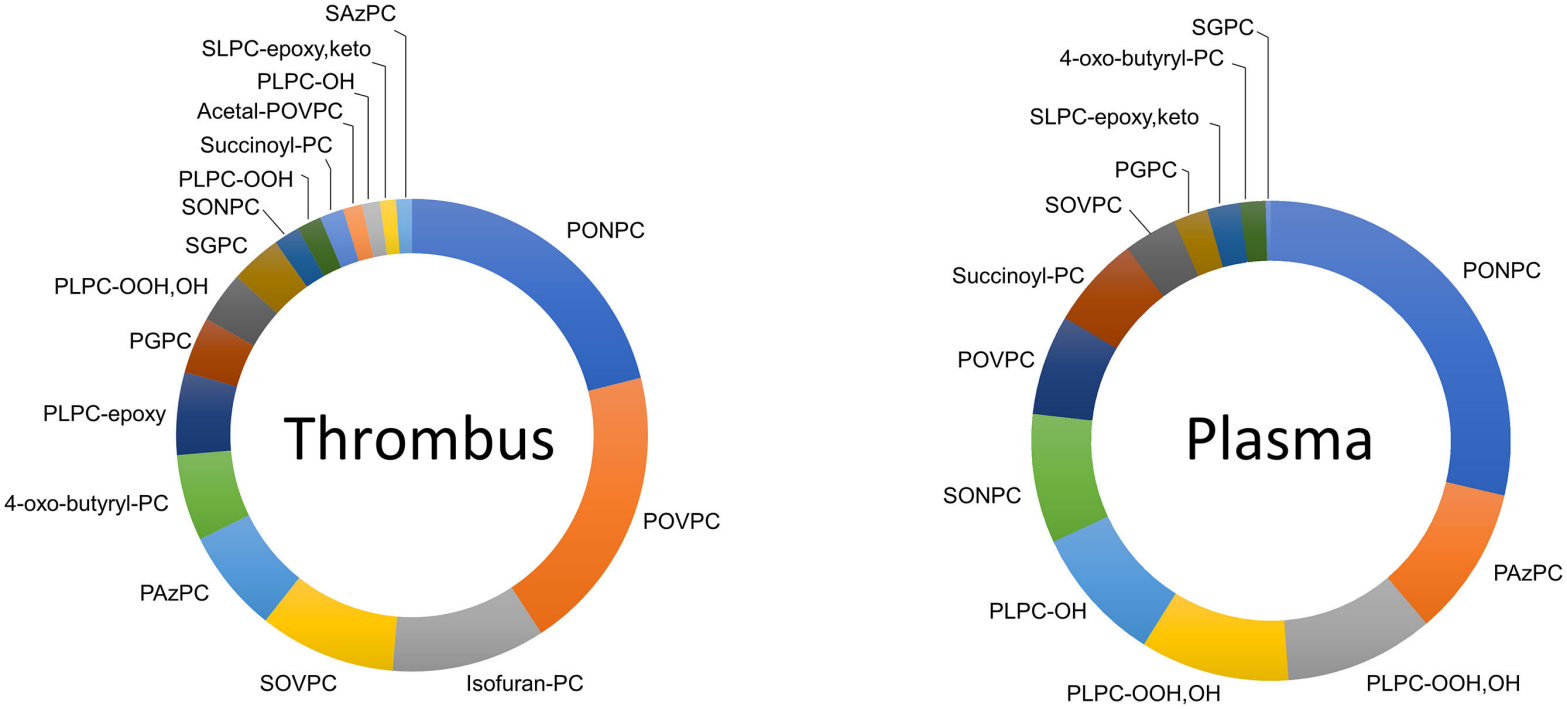
Ten most abundant OxPCs in thrombus and plasma of STEMI patients during ischemia.

Fragmented OxPCs were dominant in thrombus, which constituted 77% of total OxPCs. Fifty-four per cent and 23% of all quantified OxPCs were aldo-OxPC and acid-OxPCs, respectively. Non-fragmented OxPCs constituted only 23% of total OxPC levels in thrombus (Figure 8). PONPC and POVPC were the two most abundant OxPC in STEMI thrombus, which made 19.8 and 18.4% of total identified OxPC. The percentages of the 10 most abundant OxPC to total OxPC content of thrombus are compared with plasma levels in Figure 9.

## Discussion

There has been increasing evidence that OxPC molecules represent a novel class of bioactive lipids implicate in human pathophysiology (19). In the current study, we conducted a comprehensive analysis of OxPCs in patients presenting with STEMI and followed their OxPC levels in the acute phase and after 30 days post PCI. We were able to identify 22 OxPC species, including fragmented OxPC and non-fragmented hydroxyl/hydroperoxyl/ isoprostane OxPCs in our population. We have shown that there were increases in fragmented OxPC levels during the ischemic episode in STEMI patients compared with controls which decreased during 30 days post-MI. Moreover, the levels of POVPC and PONPC during ischemia were significantly associated with peak CK levels.

Previously, Stubiger et al. (20) conducted a targeted lipidomic analysis in plasma of 13 young patients with familial hypercholesterolemia and 7 normolipidemic individuals using LC-ESI-SRM and MALDI-QIT-TOF-MS/MS. Eight fragmented OxPC compounds containing stearyl or palmityl in their sn-1 position were identified and quantified in their population. SOVPC was the dominant OxPC in their study population. Recently, Ademowo et al. (21) applied a targeted approach to analyze OxPCs species in plasma of healthy subjects (*n* = 20), patients with chronic kidney disease (CKD) (*n* = 13), patients with periodontitis (*n* = 17) and patients with both CKD and periodontitis (*n* = 20) using LC-MS/MS. They were able to identify 12 fragmented OxPC species derived from PAPC/PLPC or SAPC/SLPC in their population. Moreover, Godzien et al. (22) used a non-targeted metabolomics approach for OxPC identification in serum of individuals with normal glucose hemostasis (*n* = 57), patients with insulin resistance (*n* = 52), prediabetics (*n* = 49) and diabetic patients (*n* = 40). They were able to identify 21 OxPC species, including 16 non-fragmented and 5 fragmented OxPC. However, only non-fragmented OxPC were quantified, as the levels of fragmented OxPC were below the limit of detection.

To find any potential roles of OxPC in I/R injury, we first compared the levels of OxPC compounds in the plasma of STEMI patients during ischemia with controls. We found that total levels of fragmented OxPCs increased significantly during the ischemic period compared with controls. However, their concentrations decreased gradually and at 30 days after reperfusion differences reached statistically significant levels (Table 3; Figure 3B). In a study by Frey et al. (23), levels of an unknown fragmented OxPC, which was measured in 5 patients undergoing coronary artery bypass grafting using liquid chromatography (after precolumn derivatization) showed a significant increase after 3 h reperfusion compared with baseline values. However, as they did not use a mass spectrometry approach, they were not able to identify the exact structure of the compound in question. Non-fragmented OxPCs, on the other hand, were significantly lower in STEMI patients during ischemia compared with controls but increased gradually during 30-day post-PPCI, although not significantly (Figure 3C).

The elevation of fragmented OxPCs and reduction of non-fragmented OxPCs levels during early I/R can be attributed to enhanced ROS productions and inflammatory response. Acute restoration of blood following reperfusion leads to the formation of ROS as a result of an imbalance between the formation of free radicals and cellular protections against them (24). Both enzymatic (xanthin oxidase and NADPH oxidase) and non-enzymatic oxidations (mitochondrial dysfunction) have been identified during myocardial I/R (14). Enhanced oxidative stress can lead to cardiomyocyte cell death by disturbing cell membrane integrity as a result of pyroptosis, necroptosis and activating mitochondria-mediated apoptosis (25).

Previous studies have assessed the levels of OxPC adducts on apo (B) and plasminogen following I/R in MI patients. Tsimikas et al. (10) used serial plasma samples collected from 8 MI patients at the time of presentation to the hospital and subsequently at 4, 30, 120, and 210 days following MI. In this study, a 54% increase was seen in OxPC on apo(B) measured by E06 antibody following MI, which reached statistical significance at 30 and 210 days following discharge. However, no such differences were observed in patients with stable angina, patients with normal coronary angiograms, and healthy controls during 7-month follow up (10). In our analysis, we measured free forms of OxPC species in plasma, but not the OxPC adducts. OxPC species, particularly fragmented forms, are bioactive and can rapidly interact with plasma proteins (9). Philippova et al. (26) also suggested that in OxPC analysis, results of LC-MS/MS and immunoassay are related but not duplicates, as they observed weak correlations between free levels of 8 OxPC species measured by LC-MS/MS and E06 antibody in plasma of patients that underwent coronary angiography. They suggested that E06 recognizes both free form and covalent adducts of OxPCs which are bound to lipoproteins, cell membranes and other plasma proteins. However, these adducts are not recognizable by the LC-MS/MS method. Besides, the E06 antibody binds not only to fragmented OxPC but also to a fraction of non-fragmented OxPCs (14).

We previously showed that fragmented aldo-OxPC, namely POVPC and PONPC enhanced significantly in both *in vitro* and *in vivo* models of I/R (14). Interestingly, in the current study, POVPC, SOVPC, PONPC, and SONPC were significantly elevated during ischemia compared with control. Their levels were also increased early in reperfusion for PONPC and SONPC. It should be mentioned that SOVPC and SONPC have similar chemical structures to POVPC and PONPC; differing only in the stearyl group at the sn-1 position instead of a palmityl group. It's been demonstrated that the active group at the sn-2 position of PL determines the bioactivity of a particular PL (27). Therefore, the active aldehyde group on SOVPC and SONPC can rapidly interact with biomolecules causing tissue injury. We have shown that introducing POVPC and PONPC to cardiomyocytes cell culture activated cell death in a dose-response manner. PONPC was the most potent OxPC, as adding 1, 2, 5, and 10 mM of PONPC resulted in significant cardiomyocyte cell death, but only high concentrations of POVPC (10 mM) induced cell death (14). PONPC and POVPC cause mitochondrial permeability through activation of Bcl-2interacting protein 3 (Bnip3), which has a critical role in cell death during cardiomyocytes I/R injury. We have recently shown that treatment of cardiomyocytes with aldo-OxPCs, namely, POVPC and PONPC results in potent ferroptotic cell death. In this study, both POVPC and PONPC suppressed glutathione peroxidase-4 (GPx4), an enzyme implicating in ferroptosis. Finally, treatment of cardiomyocyte with ferrostatin-1, which is an inhibitor of ferroptosis suppressed cell death induced by OxPCs (12).

In the current study, POVPC and PONPC levels during the ischemic period were significantly associated with higher peak CK levels (Figure 7). Previous studies confirmed that elevated CK levels are associated with larger infarct size (28) and higher mortality (29). Moreover, the E06 antibody can inhibit cell death during I/R through the deactivation of aldo-OxPCs. Taken together, the correlation between OxPC species, namely, PONPC and POVPC with CK levels can suggest their roles in I/R injury following I/R.

Based on the finding of our study, we believe that aldo-OxPCs are mediators of I/R injury. Not only these compounds rise and fall with ischemia and reperfusion, but they predict increased CK levels, a marker of total infarct size. However, we did not see any linear correlations between CK and any OxPC species. This is likely a result of our small sample size given that the trend was seen for Troponins.

In the current study, we also determined the OxPC profile from thrombectomy samples from patients presenting with STEMI. This showed that fragmented OxPCs were the largest proportion of OxPC species in the recovered thrombotic plaque material, as 77% of total OxPCs were fragmented species (Figure 8). Aldo-OxPCs were 2-time higher than acid-OxPCs, and PONPC and POVPC constituted 38.5% of the total identified OxPCs on thrombus. We have previously shown that the plaque material recovered at the time of angioplasty from carotid, saphenous vein grafts, and renal artery during PCI are rich in OxPC with a large proportion representing fragmented species. PONPC and POVPC were the most common compound in iatrogenic plaque, which made up 50% of the identified OxPCs (30).

As it's shown in Figure 8, the OxPC profile of thrombus is different from plasma during I/R. While 77% of total OxPCs is fragmented species in thrombus, it is only 35% of the total OxPC of plasma during the ischemic time. The composition of OxPCs in the plasma at the time of STEMI presentation was different from the composition of coronary thrombus. Thrombus had a higher proportion of POVPC and SOVPC than plasma, although they both have high levels of PONPC (Figure 9). This indicates that plasma OxPCs are not explained by OxPCs from thrombus, rather, thrombus and plasma have different OxPC profiles that cannot be explained by a single common pathway.

The current study has several potential limitations: First, our sample size was relatively small considering the high prevalence of comorbidities and medication use in the STEMI population. However, serial blood sampling helped to lessen inter-individual variability and clinical confounders. Moreover, due to the small sample size, we were not able to compare the effects of sex, age, ethnicity, etc in our population. Hence, it is important to validate the findings of this study in a relatively larger cohort study. Second, considering there are no available commercial standards for all individual OxPC species, the reported concentrations are relative rather than absolute.

In summary, this study showed that biologically active fragmented OxPC increased in patients presenting with STEMI when compared to controls. PONPC concentrations were subsequently increased after primary PCI resulting in reperfusion. Moreover, levels of POVPC and PONPC were also associated with peak CK levels. Since fragmented aldo-OxPCs are potent stimulators for cardiomyocyte cell death, therapeutics that inhibit their activities can result in a novel therapeutic pathway for myocardial salvage for patients undergoing reperfusion therapy.

## Data Availability Statement

The original contributions presented in the study are included in the article/Supplementary Material, further inquiries can be directed to the corresponding author/s.

## Ethics Statement

The studies involving human participants were reviewed and approved by University of Manitoba Biomedical Research Ethics Board. The patients/participants provided their written informed consent to participate in this study.

## Author Contributions

AR and ZS contributed to conception and design of the study. ZS, AE, and MR performed the mass spectrometric and statistical analysis. ZS wrote the first draft of the manuscript. ZS, AR, DA, MR, and AS wrote sections of the manuscript. All authors contributed to manuscript revision, read, and approved the submitted version.

## Funding

This work was supported by the National Institutes of Health (NIH) and Research Manitoba.

## Conflict of Interest

The authors declare that the research was conducted in the absence of any commercial or financial relationships that could be construed as a potential conflict of interest.

## Publisher's Note

All claims expressed in this article are solely those of the authors and do not necessarily represent those of their affiliated organizations, or those of the publisher, the editors and the reviewers. Any product that may be evaluated in this article, or claim that may be made by its manufacturer, is not guaranteed or endorsed by the publisher.

## Abbreviations

MI: Myocardial infarction
STEMI: ST-segment elevation myocardial infarction
I/R: Ischemia/reperfusion
ROS: Reactive oxygen species
OxPC: Oxidized phosphatidylcholine
PL: Phospholipid
OxPL: Oxidized phospholipid
PAPC: 1-palmitoyl-2-arachidonoyl-sn-glycerol-3-phosphatidylcholine
PLPC: 1-Palmitoyl-2-lauroyl-sn-glycero-3-phosphocholine
SAPC: 1-stearoyl-2-arachidonoyl-sn-glycero-phosphocholine
SLPC: 1-stearoyl-2-linoleoyl-sn-glycero-phosphocholine
POVPC: 1-palmitoyl-2-(5′-oxo-valeroyl)-sn-glycero-3-phosphocholine
PONPC: 1-palmitoyl-2-(9-oxo-nonanoyl)-sn-glycero-3-phosphocholine
SOVPC: 1-stearoyl-2-(5′-oxo-valeroyl)-sn-glycero-3-phosphocholine
SONPC: 1-stearoyl-2-(9-oxo-nonanoyl)-sn-glycero-3-phosphocholine.
