# Supplementary material for: Increase in Plasma Oxidized Phosphatidylcholines (OxPCs) in Patients Presenting With ST-Elevation Myocardial Infarction (STEMI)
Source: Front Med (Lausanne). 2021 Dec 1;8:716944. doi: 10.3389/fmed.2021.716944 (PMC8671696; doi:10.3389/fmed.2021.716944)
Supplement: Supplementary file 1 [file Data_Sheet_1.docx]

**Increase in plasma oxidized phosphatidylcholines (OxPCs) in patients presenting with ST-Elevation Myocardial Infarction (STEMI)**

Zahra Solati^1,2^, Arun Surendran^1,2^, Andrea Edel^1,2^, Marynia Roznik^3^, David Allen^4^, and Amir Ravandi^1,2,3,4^

^1^Cardiovascular Lipidomics Laboratory, St. Boniface Hospital, Albrechtsen Research Centre,

^2^Department of Physiology and Pathophysiology, Rady Faculty of Health Sciences, University of Manitoba,

^3^Department of Medicine, Rady Faculty of Health Sciences, University of Manitoba,

^4^Section of Cardiology, Department of Medicine, Rady Faculty of Health Sciences, University of Manitoba

**Running title:** Oxidized phosphatidylcholines and myocardial reperfusion

**Corresponding author**

Dr. Amir Ravandi

Cardiovascular Lipidomics Laboratory,

St. Boniface Hospital Albrechtsen Research Centre,

351 Tache Ave,

Winnipeg, MB Canada R2H 2A6

Phone.204-235-3206 and 204-235-3414

Fax.204-235-0793 and 204-235-0793

Email: [aravandi@sbgh.mb.ca](mailto:aravandi@sbgh.mb.ca)

**Materials:**

Phospholipid and oxidized phospholipids including 1,2-dinonanoyl-sn-glycero-3-phosphocholine (09:0 PC), 1-palmitoyl-2-linoleoyl-sn-glycero-3-phosphocholine (PLPC), 1-palmitoyl-2-arachidonoyl-sn-glycero-3-phosphocholine (PAPC), 1-stearoyl-2-linoleoyl-sn-glycero-3-phosphocholine (SLPC), 1-stearoyl-2-arachidonoyl-sn-glycero-3-phosphocholine (SAPC), 1-palmitoyl-2-docosahexaenoyl-sn-glycero-3-phosphocholine (PDHPC), 1-palmitoyl-2-(5'-oxo-valeroyl)-sn-glycero-3-phosphocholine (POVPC), 1-palmitoyl-2-azelaoyl-sn-glycero-3-phosphocholine (PAzPC) and 1-palmitoyl-2-(9'-oxo-nonanoyl)-sn-glycero-3-phosphocholine (PONPC), and 1-palmitoyl-2-glutaryl-sn-glycero-3-phosphocholine (PGPC) were obtained from Avanti Polar Lipids (Alabaster, AL, USA). 1-palmitoyl-2-(5’-keto-6’-octenedioyl)-sn-glycero-3-phosphocholine (KOdiA-PC) and 1-palmitoyl-2(4’-keto-dodec-3’-ene-dioyl)-sn-glycero-3-phosphocholine (KDdiA-PC) were purchased from Cayman Chemicals (Ann Arbor, Michigan, USA). Analytical grade chloroform, methanol, and formic acid were purchased from Fisher-Scientific (Hampton, NH). Mobile phase solvents including acetonitrile, isopropanol, and water (LC/MS grade) were purchased from Fisher-Scientific.

**Supplemetary Fig I: MRM chromatogram of OxPC standards:**
Identification of commercial OxPC standards by reversed-phase LC-MS/MS


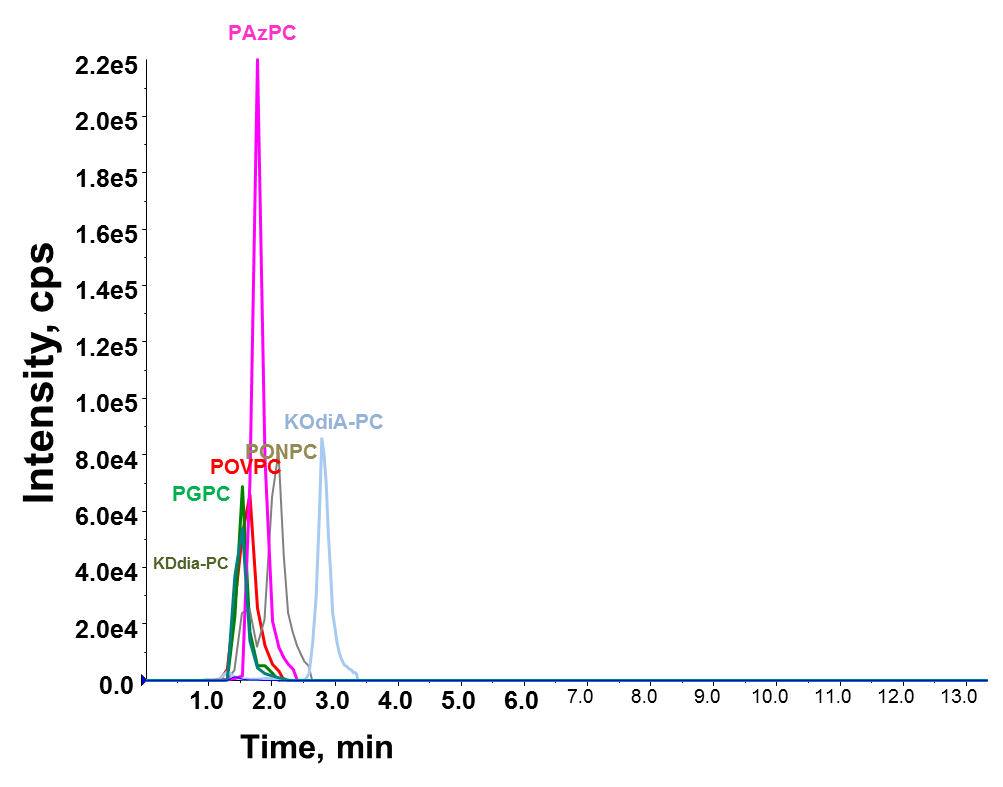


**STable I: List of OxPC compounds identified by reversed-phase LC/MS/MS**

OxPC species produced by air oxidation of PAPC, PLPC, SAPC, SLPC, PDHPC, and SDHPC using HPLC-MS/MS

| **Q1 Mass (Da)** | **Q3 Mass (Da)** | **Retention time** | **OxPC Compound** | **DP (Volts)** | **EP (Volts)** | **CE (Volts)** | **CXP (Volts)** |
| --- | --- | --- | --- | --- | --- | --- | --- |
| 538.6 | 184.3 | 1.29 | **di-9:0-PC (internal standard)** | 125 | 10 | 53 | 9 |
| 580.6 | 184.3 | 1.45 | **4-oxo-butyryl-PC** | 125 | 10 | 53 | 9 |
| 594.6 | 184.3 | 1.48 | **POVPC** | 125 | 10 | 53 | 9 |
| 596.6 | 184.3 | 1.39 | **Succinoyl-PC** | 125 | 10 | 53 | 9 |
| 610.6 | 184.3 | 1.4 | **PGPC** | 125 | 10 | 53 | 9 |
| 622.6 | 184.3 | 2.16 | **SOVPC** | 125 | 10 | 53 | 9 |
| 634.6 | 184.3 | 1.39 | **KOHA-PC** | 125 | 10 | 53 | 9 |
| 636.6 | 184.3 | 1.75 | **8-oxo-octanoyl-PPC (PLPC)** | 125 | 10 | 53 | 9 |
| 638.6 | 184.3 | 1.98 | **SGPC** | 125 | 10 | 53 | 9 |
| 640.6 | 184.3 | 1.37 | **Acetal-POVPC** | 125 | 10 | 53 | 9 |
| 648.6 | 184.3 | 1.44 | **KOOA-PC** | 125 | 10 | 53 | 9 |
| 650.6 | 184.3 | 1.98 | **PONPC** | 125 | 10 | 53 | 9 |
| 664.6 | 184.3 | 2.72 | **KOdiA-PC** | 125 | 10 | 53 | 9 |
| 666.6 | 184.3 | 1.7 | **PAzPC** | 125 | 10 | 53 | 9 |
| 676.6 | 184.3 | 2.1 | **KOOA-SPC 11-oxo-9-undecenoyl-PPC** | 125 | 10 | 53 | 9 |
| 678.6 | 184.3 | 3.15 | **SONPC** | 125 | 10 | 53 | 9 |
| 688.6 | 184.3 | 1.46 | **Furyloctanoyl-PC** | 125 | 10 | 53 | 9 |
| 694.6 | 184.3 | 2.56 | **SAzPC** | 125 | 10 | 53 | 9 |
| 696.6 | 184.3 | 1.54 | **Acetal-PONPC** | 125 | 10 | 53 | 9 |
| 704.6 | 184.3 | 1.74 | **KODA-PPC (PLPC)8-OOH-9-oxo-nonanoyl-PPC (PLPC)** | 125 | 10 | 53 | 9 |
| 706.6 | 184.3 | 1.62 | **HODA-PPC** | 125 | 10 | 53 | 9 |
| 716.6 | 184.3 | 2.08 | **Furyloctanoyl-PC** | 125 | 10 | 53 | 9 |
| 720.6 | 184.3 | 1.51 | **KDdiA-PC** | 125 | 10 | 53 | 9 |
| 722.6 | 184.3 | 1.51 | **HDdiA-PPC** | 125 | 10 | 53 | 9 |
| 724.6 | 184.3 | 2.18 | **Acetal-SONPC** | 125 | 10 | 53 | 9 |
| 734.6 | 184.3 | 2.44 | **HODA-SPC** | 125 | 10 | 53 | 9 |
| 748.6 | 184.3 | 2.11 | **KDiA-SPC** | 125 | 10 | 53 | 9 |
| 750.6 | 184.3 | 2.1 | **HDiA-PC** | 125 | 10 | 53 | 9 |
| 772.6 | 184.3 | 5.57 | **PLPC-keto** | 125 | 10 | 53 | 9 |
| 774.6 | 184.3 | 5.48 | **PLPC-OH** | 125 | 10 | 53 | 9 |
| 788.6 | 184.3 | 4.53 | **PLPC-epoxy,ketoPLPC-OH,keto** | 125 | 10 | 53 | 9 |
| 790.6 | 184.3 | 4.28 | **PLPC-OOH** | 125 | 10 | 53 | 9 |
| 796.6 | 184.3 | 5.88 | **PAPC-keto** | 125 | 10 | 53 | 9 |
| 798.6 | 184.3 | 7.09 | **PAPC-OH** | 125 | 10 | 53 | 9 |
| 800.6 | 184.3 | 6.51 | **SLPC-keto** | 125 | 10 | 53 | 9 |
| 802.6 | 184.3 | 6.7 | **SLPC-OH** | 125 | 10 | 53 | 9 |
| 806.6 | 184.3 | 6.74 | **PLPC-OOH,OH** | 125 | 10 | 53 | 9 |
| 808.6 | 184.3 | 6.71 | **PLPC-diOH,epoxy** | 125 | 10 | 53 | 9 |
| 814.6 | 184.3 | 6.73 | **PAPC-OOH** | 125 | 10 | 53 | 9 |
| 816.6 | 184.3 | 6 | **SLPC-epoxy,keto** | 125 | 10 | 53 | 9 |
| 818.6 | 184.3 | 5.7 | **SLPC-OOH** | 125 | 10 | 53 | 9 |
| 824.6 | 184.3 | 6.83 | **SAPC-keto** | 125 | 10 | 53 | 9 |
| 826.6 | 184.3 | 8.19 | **SAPC-OH** | 125 | 10 | 53 | 9 |
| 832.6 | 184.3 | 7.6 | **isoPGF2alpha-PC** | 125 | 10 | 53 | 9 |
| 834.6 | 184.3 | 5.58 | **SLPC-OOH,OH** | 125 | 10 | 53 | 9 |
| 836.6 | 184.3 | 7.31 | **SLPC-triOH** | 125 | 10 | 53 | 9 |
| 842.6 | 184.3 | 6.98 | **SAPC-OOH** | 125 | 10 | 53 | 9 |
| 856.6 | 184.3 | 4.83 | **SEIPC** | 125 | 10 | 53 | 9 |
| 860.6 | 184.3 | 8.13 | **isoPGF2alpha-PC** | 125 | 10 | 53 | 9 |
| 872.6 | 184.3 | 4.32 | **SAPC-OOH,OH,keto** | 125 | 10 | 53 | 9 |
| 882.6 | 184.3 | 6.93 | **SLPC-triOOH** | 125 | 10 | 53 | 9 |
